# Supplementary material for: Resolving the full spectrum of human genome variation using Linked-Reads
Source: Genome Res. 2019 Apr;29(4):635–45. doi: 10.1101/gr.234443.118 (PMC6442396; doi:10.1101/gr.234443.118)
Supplement: Supplemental Material [file supp_gr.234443.118_Supplemental_longranger-2.2.zip › longranger-2.2.tar.bz2!/lr2.2-public/lib/stan/cmdstan/stan/doc/api/html/beta__binomial_8hpp_source.html]

Stan: src/stan/prob/distributions/univariate/discrete/beta\_binomial.hpp Source File


|  |  |
| --- | --- |
|  | Stan  2.5.0  probability, sampling & optimization |


- Main Page
- Related Pages
- Namespaces
- Classes
- Files

- File List
- File Members

All Classes Namespaces Files Functions Variables Typedefs Enumerations Enumerator Friends Macros Pages

- src
- stan
- prob
- distributions
- univariate
- discrete

beta\_binomial.hpp

Go to the documentation of this file.

1 #ifndef STAN\_\_PROB\_\_DISTRIBUTIONS\_\_UNIVARIATE\_\_DISCRETE\_\_BETA\_BINOMIAL\_HPP

2 #define STAN\_\_PROB\_\_DISTRIBUTIONS\_\_UNIVARIATE\_\_DISCRETE\_\_BETA\_BINOMIAL\_HPP

3

4 #include <stan/prob/distributions/univariate/discrete/binomial.hpp>

5 #include <stan/prob/distributions/univariate/continuous/beta.hpp>

6

7 #include <stan/agrad/partials\_vari.hpp>

8 #include <stan/math/error\_handling.hpp>

9 #include <stan/math/constants.hpp>

10 #include <stan/math/functions/lbeta.hpp>

11 #include <stan/math/functions/value\_of.hpp>

12 #include <stan/meta/traits.hpp>

13 #include <stan/prob/traits.hpp>

14 #include <stan/prob/constants.hpp>

15 #include <stan/prob/internal\_math.hpp>

16

17 #include <stan/math/functions/binomial\_coefficient\_log.hpp>

18

19 namespace stan {

20

21  namespace prob {

22

23  // BetaBinomial(n|alpha,beta) [alpha > 0; beta > 0; n >= 0]

24  template <bool propto,

25  typename T\_n, typename T\_N,

26  typename T\_size1, typename T\_size2>

27  typename return\_type<T\_size1,T\_size2>::type

28  beta\_binomial\_log(const T\_n& n,

29  const T\_N& N,

30  const T\_size1& alpha,

31  const T\_size2& beta) {

32  static const char\* function = "stan::prob::beta\_binomial\_log(%1%)";

33

34  using stan::math::check\_positive\_finite;

35  using stan::math::check\_nonnegative;

36  using stan::math::value\_of;

37  using stan::math::check\_consistent\_sizes;

38  using stan::prob::include\_summand;

39

40  // check if any vectors are zero length

41  if (!(stan::length(n)

42  && stan::length(N)

43  && stan::length(alpha)

44  && stan::length(beta)))

45  return 0.0;

46

47  double logp(0.0);

48  check\_nonnegative(function, N, "Population size parameter", &logp);

49  check\_positive\_finite(function, alpha,

50  "First prior sample size parameter",

51  &logp);

52  check\_positive\_finite(function, beta,

53  "Second prior sample size parameter",

54  &logp);

55  check\_consistent\_sizes(function,

56  n,N,alpha,beta,

57  "Successes variable",

58  "Population size parameter",

59  "First prior sample size parameter",

60  "Second prior sample size parameter",

61  &logp);

62

63  // check if no variables are involved and prop-to

64  if (!include\_summand<propto,T\_size1,T\_size2>::value)

65  return 0.0;

66

67  VectorView<const T\_n> n\_vec(n);

68  VectorView<const T\_N> N\_vec(N);

69  VectorView<const T\_size1> alpha\_vec(alpha);

70  VectorView<const T\_size2> beta\_vec(beta);

71  size\_t size = max\_size(n, N, alpha, beta);

72

73  for (size\_t i = 0; i < size; i++) {

74  if (n\_vec[i] < 0 || n\_vec[i] > N\_vec[i])

75  return LOG\_ZERO;

76  }

77

78  using stan::math::lbeta;

79  using stan::math::binomial\_coefficient\_log;

80  using boost::math::digamma;

81

82  DoubleVectorView<include\_summand<propto>::value,

83  is\_vector<T\_n>::value || is\_vector<T\_N>::value>

84  normalizing\_constant(max\_size(N,n));

85  for (size\_t i = 0; i < max\_size(N,n); i++)

86  if (include\_summand<propto>::value)

87  normalizing\_constant[i]

88  = binomial\_coefficient\_log(N\_vec[i],n\_vec[i]);

89

90  DoubleVectorView<include\_summand<propto,T\_size1,T\_size2>::value,

91  is\_vector<T\_n>::value || is\_vector<T\_N>::value

92  || is\_vector<T\_size1>::value || is\_vector<T\_size2>::value>

93  lbeta\_numerator(size);

94  for (size\_t i = 0; i < size; i++)

95  if (include\_summand<propto,T\_size1,T\_size2>::value)

96  lbeta\_numerator[i] = lbeta(n\_vec[i] + value\_of(alpha\_vec[i]),

97  N\_vec[i] - n\_vec[i]

98  + value\_of(beta\_vec[i]));

99  DoubleVectorView<include\_summand<propto,T\_size1,T\_size2>::value,

100  is\_vector<T\_size1>::value || is\_vector<T\_size2>::value>

101  lbeta\_denominator(max\_size(alpha,beta));

102 for (size\_t i = 0; i < max\_size(alpha,beta); i++)

103  if (include\_summand<propto,T\_size1,T\_size2>::value)

104  lbeta\_denominator[i] = lbeta(value\_of(alpha\_vec[i]),

105  value\_of(beta\_vec[i]));

106

107 DoubleVectorView<!is\_constant\_struct<T\_size1>::value,

108  is\_vector<T\_n>::value || is\_vector<T\_size1>::value>

109  digamma\_n\_plus\_alpha(max\_size(n,alpha));

110  for (size\_t i = 0; i < max\_size(n,alpha); i++)

111  if (!is\_constant\_struct<T\_size1>::value)

112  digamma\_n\_plus\_alpha[i]

113  = digamma(n\_vec[i] + value\_of(alpha\_vec[i]));

114

115  DoubleVectorView<!is\_constant\_struct<T\_size1>::value

116  || !is\_constant\_struct<T\_size2>::value,

117  is\_vector<T\_N>::value

118  || is\_vector<T\_size1>::value

119  || is\_vector<T\_size1>::value>

120  digamma\_N\_plus\_alpha\_plus\_beta(max\_size(N,alpha,beta));

121  for (size\_t i = 0; i < max\_size(N,alpha,beta); i++)

122  if (!is\_constant\_struct<T\_size1>::value

123  || !is\_constant\_struct<T\_size2>::value)

124  digamma\_N\_plus\_alpha\_plus\_beta[i]

125  = digamma(N\_vec[i] + value\_of(alpha\_vec[i]) + value\_of(beta\_vec[i]));

126

127  DoubleVectorView<!is\_constant\_struct<T\_size1>::value

128  || !is\_constant\_struct<T\_size2>::value,

129  is\_vector<T\_size1>::value

130  || is\_vector<T\_size1>::value>

131  digamma\_alpha\_plus\_beta(max\_size(alpha,beta));

132  for (size\_t i = 0; i < max\_size(alpha,beta); i++)

133  if (!is\_constant\_struct<T\_size1>::value

134  || !is\_constant\_struct<T\_size2>::value)

135  digamma\_alpha\_plus\_beta[i]

136  = digamma(value\_of(alpha\_vec[i]) + value\_of(beta\_vec[i]));

137

138  DoubleVectorView<!is\_constant\_struct<T\_size1>::value,

139  is\_vector<T\_size1>::value>

140  digamma\_alpha(length(alpha));

141  for (size\_t i = 0; i < length(alpha); i++)

142  if (!is\_constant\_struct<T\_size1>::value)

143  digamma\_alpha[i] = digamma(value\_of(alpha\_vec[i]));

144

145  DoubleVectorView<!is\_constant\_struct<T\_size2>::value,

146  is\_vector<T\_size2>::value>

147  digamma\_beta(length(beta));

148  for (size\_t i = 0; i < length(beta); i++)

149  if (!is\_constant\_struct<T\_size2>::value)

150  digamma\_beta[i] = digamma(value\_of(beta\_vec[i]));

151

152  agrad::OperandsAndPartials<T\_n,T\_N,T\_size1,T\_size2>

153  operands\_and\_partials(n,N,alpha,beta);

154  for (size\_t i = 0; i < size; i++) {

155  if (include\_summand<propto>::value)

156  logp += normalizing\_constant[i];

157  if (include\_summand<propto,T\_size1,T\_size2>::value)

158  logp += lbeta\_numerator[i]

159  - lbeta\_denominator[i];

160

161  if (!is\_constant\_struct<T\_size1>::value)

162  operands\_and\_partials.d\_x3[i]

163  += digamma\_n\_plus\_alpha[i]

164  - digamma\_N\_plus\_alpha\_plus\_beta[i]

165  + digamma\_alpha\_plus\_beta[i]

166  - digamma\_alpha[i];

167  if (!is\_constant\_struct<T\_size2>::value)

168  operands\_and\_partials.d\_x4[i]

169  += digamma(value\_of(N\_vec[i]-n\_vec[i]+beta\_vec[i]))

170  - digamma\_N\_plus\_alpha\_plus\_beta[i]

171  + digamma\_alpha\_plus\_beta[i]

172  - digamma\_beta[i];

173  }

174  return operands\_and\_partials.to\_var(logp);

175  }

176

177  template <typename T\_n,

178  typename T\_N,

179  typename T\_size1,

180  typename T\_size2>

181  typename return\_type<T\_size1,T\_size2>::type

182  beta\_binomial\_log(const T\_n& n, const T\_N& N,

183  const T\_size1& alpha, const T\_size2& beta) {

184  return beta\_binomial\_log<false>(n,N,alpha,beta);

185  }

186

187  // Beta-Binomial CDF

188  template <typename T\_n, typename T\_N,

189  typename T\_size1, typename T\_size2>

190  typename return\_type<T\_size1,T\_size2>::type

191  beta\_binomial\_cdf(const T\_n& n, const T\_N& N, const T\_size1& alpha,

192  const T\_size2& beta) {

193

194  static const char\* function = "stan::prob::beta\_binomial\_cdf(%1%)";

195

196  using stan::math::check\_positive\_finite;

197  using stan::math::check\_nonnegative;

198  using stan::math::value\_of;

199  using stan::math::check\_consistent\_sizes;

200  using stan::prob::include\_summand;

201

202  // Ensure non-zero argument lengths

203  if (!(stan::length(n) && stan::length(N) && stan::length(alpha)

204  && stan::length(beta)))

205  return 1.0;

206

207  double P(1.0);

208

209  // Validate arguments

210  check\_nonnegative(function, N, "Population size parameter", &P);

211  check\_positive\_finite(function, alpha, "First prior sample size parameter",

212  &P);

213  check\_positive\_finite(function, beta, "Second prior sample size parameter",

214  &P);

215  check\_consistent\_sizes(function,

216  n, N, alpha, beta,

217  "Successes variable",

218  "Population size parameter",

219  "First prior sample size parameter",

220  "Second prior sample size parameter",

221  &P);

222

223  // Wrap arguments in vector views

224  VectorView<const T\_n> n\_vec(n);

225  VectorView<const T\_N> N\_vec(N);

226  VectorView<const T\_size1> alpha\_vec(alpha);

227  VectorView<const T\_size2> beta\_vec(beta);

228  size\_t size = max\_size(n, N, alpha, beta);

229

230  // Compute vectorized CDF and gradient

231  using boost::math::lgamma;

232  using boost::math::digamma;

233

234  agrad::OperandsAndPartials<T\_size1, T\_size2>

235  operands\_and\_partials(alpha, beta);

236

237  // Explicit return for extreme values

238  // The gradients are technically ill-defined, but treated as zero

239  for (size\_t i = 0; i < stan::length(n); i++) {

240  if (value\_of(n\_vec[i]) <= 0)

241  return operands\_and\_partials.to\_var(0.0);

242  }

243

244  for (size\_t i = 0; i < size; i++) {

245  // Explicit results for extreme values

246  // The gradients are technically ill-defined, but treated as zero

247  if (value\_of(n\_vec[i]) >= value\_of(N\_vec[i])) {

248  continue;

249  }

250

251  const double n\_dbl = value\_of(n\_vec[i]);

252  const double N\_dbl = value\_of(N\_vec[i]);

253  const double alpha\_dbl = value\_of(alpha\_vec[i]);

254  const double beta\_dbl = value\_of(beta\_vec[i]);

255

256  const double mu = alpha\_dbl + n\_dbl + 1;

257  const double nu = beta\_dbl + N\_dbl - n\_dbl - 1;

258

259  const double F = stan::math::F32(1, mu, -N\_dbl + n\_dbl + 1, n\_dbl + 2,

260  1 - nu, 1);

261

262  double C = lgamma(nu) - lgamma(N\_dbl - n\_dbl);

263  C += lgamma(mu) - lgamma(n\_dbl + 2);

264  C += lgamma(N\_dbl + 2) - lgamma(N\_dbl + alpha\_dbl + beta\_dbl);

265  C = std::exp(C);

266

267  C \*= F / boost::math::beta(alpha\_dbl, beta\_dbl);

268  C /= N\_dbl + 1;

269

270  const double Pi = 1 - C;

271

272  P \*= Pi;

273

274  double dF[6];

275  double digammaOne = 0;

276  double digammaTwo = 0;

277

278  if ( (!is\_constant\_struct<T\_size1>::value)

279  || (!is\_constant\_struct<T\_size2>::value) ) {

280  digammaOne = digamma(mu + nu);

281  digammaTwo = digamma(alpha\_dbl + beta\_dbl);

282  stan::math::gradF32(dF, 1, mu, -N\_dbl + n\_dbl + 1, n\_dbl + 2,

283  1 - nu, 1);

284  }

285  if (!is\_constant\_struct<T\_size1>::value) {

286  const double g

287  = - C \* (digamma(mu) - digammaOne + dF[1] / F

288  - digamma(alpha\_dbl) + digammaTwo);

289  operands\_and\_partials.d\_x1[i]

290  += g / Pi;

291  }

292  if (!is\_constant\_struct<T\_size2>::value) {

293  const double g

294  = - C \* (digamma(nu) - digammaOne - dF[4] / F - digamma(beta\_dbl)

295  + digammaTwo);

296  operands\_and\_partials.d\_x2[i]

297  += g / Pi;

298  }

299  }

300

301  if (!is\_constant\_struct<T\_size1>::value)

302  for(size\_t i = 0; i < stan::length(alpha); ++i)

303  operands\_and\_partials.d\_x1[i] \*= P;

304  if (!is\_constant\_struct<T\_size2>::value)

305  for(size\_t i = 0; i < stan::length(beta); ++i)

306  operands\_and\_partials.d\_x2[i] \*= P;

307

308  return operands\_and\_partials.to\_var(P);

309  }

310

311  template <typename T\_n, typename T\_N,

312  typename T\_size1, typename T\_size2>

313  typename return\_type<T\_size1,T\_size2>::type

314  beta\_binomial\_cdf\_log(const T\_n& n, const T\_N& N, const T\_size1& alpha,

315  const T\_size2& beta) {

316

317  static const char\* function = "stan::prob::beta\_binomial\_cdf\_log(%1%)";

318

319  using stan::math::check\_positive\_finite;

320  using stan::math::check\_nonnegative;

321  using stan::math::value\_of;

322  using stan::math::check\_consistent\_sizes;

323  using stan::prob::include\_summand;

324

325  // Ensure non-zero argument lengths

326  if (!(stan::length(n) && stan::length(N) && stan::length(alpha)

327  && stan::length(beta)))

328  return 0.0;

329

330  double P(0.0);

331

332  // Validate arguments

333  check\_nonnegative(function, N, "Population size parameter", &P);

334  check\_positive\_finite(function, alpha, "First prior sample size parameter",

335  &P);

336  check\_positive\_finite(function, beta, "Second prior sample size parameter",

337  &P);

338  check\_consistent\_sizes(function,

339  n, N, alpha, beta,

340  "Successes variable",

341  "Population size parameter",

342  "First prior sample size parameter",

343  "Second prior sample size parameter",

344  &P);

345

346  // Wrap arguments in vector views

347  VectorView<const T\_n> n\_vec(n);

348  VectorView<const T\_N> N\_vec(N);

349  VectorView<const T\_size1> alpha\_vec(alpha);

350  VectorView<const T\_size2> beta\_vec(beta);

351  size\_t size = max\_size(n, N, alpha, beta);

352

353  // Compute vectorized cdf\_log and gradient

354  using boost::math::lgamma;

355  using boost::math::digamma;

356

357  agrad::OperandsAndPartials<T\_size1, T\_size2>

358  operands\_and\_partials(alpha, beta);

359

360  // Explicit return for extreme values

361  // The gradients are technically ill-defined, but treated as neg infinity

362  for (size\_t i = 0; i < stan::length(n); i++) {

363  if (value\_of(n\_vec[i]) <= 0)

364  return operands\_and\_partials.to\_var(stan::math::negative\_infinity());

365  }

366

367  for (size\_t i = 0; i < size; i++) {

368  // Explicit results for extreme values

369  // The gradients are technically ill-defined, but treated as zero

370  if (value\_of(n\_vec[i]) >= value\_of(N\_vec[i])) {

371  continue;

372  }

373

374  const double n\_dbl = value\_of(n\_vec[i]);

375  const double N\_dbl = value\_of(N\_vec[i]);

376  const double alpha\_dbl = value\_of(alpha\_vec[i]);

377  const double beta\_dbl = value\_of(beta\_vec[i]);

378

379  const double mu = alpha\_dbl + n\_dbl + 1;

380  const double nu = beta\_dbl + N\_dbl - n\_dbl - 1;

381

382  const double F = stan::math::F32(1, mu, -N\_dbl + n\_dbl + 1, n\_dbl + 2,

383  1 - nu, 1);

384

385  double C = lgamma(nu) - lgamma(N\_dbl - n\_dbl);

386  C += lgamma(mu) - lgamma(n\_dbl + 2);

387  C += lgamma(N\_dbl + 2) - lgamma(N\_dbl + alpha\_dbl + beta\_dbl);

388  C = std::exp(C);

389

390  C \*= F / boost::math::beta(alpha\_dbl, beta\_dbl);

391  C /= N\_dbl + 1;

392

393  const double Pi = 1 - C;

394

395  P += log(Pi);

396

397  double dF[6];

398  double digammaOne = 0;

399  double digammaTwo = 0;

400

401  if ( (!is\_constant\_struct<T\_size1>::value)

402  || (!is\_constant\_struct<T\_size2>::value) ) {

403  digammaOne = digamma(mu + nu);

404  digammaTwo = digamma(alpha\_dbl + beta\_dbl);

405  stan::math::gradF32(dF, 1, mu, -N\_dbl + n\_dbl + 1, n\_dbl + 2,

406  1 - nu, 1);

407  }

408  if (!is\_constant\_struct<T\_size1>::value) {

409  const double g

410  = - C \* (digamma(mu) - digammaOne + dF[1] / F

411  - digamma(alpha\_dbl) + digammaTwo);

412  operands\_and\_partials.d\_x1[i] += g / Pi;

413  }

414  if (!is\_constant\_struct<T\_size2>::value) {

415  const double g

416  = - C \* (digamma(nu) - digammaOne - dF[4] / F - digamma(beta\_dbl)

417  + digammaTwo);

418  operands\_and\_partials.d\_x2[i] += g / Pi;

419  }

420  }

421

422  return operands\_and\_partials.to\_var(P);

423  }

424

425  template <typename T\_n, typename T\_N,

426  typename T\_size1, typename T\_size2>

427  typename return\_type<T\_size1,T\_size2>::type

428  beta\_binomial\_ccdf\_log(const T\_n& n, const T\_N& N, const T\_size1& alpha,

429  const T\_size2& beta) {

430

431  static const char\* function = "stan::prob::beta\_binomial\_ccdf\_log(%1%)";

432

433  using stan::math::check\_positive\_finite;

434  using stan::math::check\_nonnegative;

435  using stan::math::value\_of;

436  using stan::math::check\_consistent\_sizes;

437  using stan::prob::include\_summand;

438

439  // Ensure non-zero argument lengths

440  if (!(stan::length(n) && stan::length(N) && stan::length(alpha)

441  && stan::length(beta)))

442  return 0.0;

443

444  double P(0.0);

445

446  // Validate arguments

447  check\_nonnegative(function, N, "Population size parameter", &P);

448  check\_positive\_finite(function, alpha, "First prior sample size parameter",

449  &P);

450  check\_positive\_finite(function, beta, "Second prior sample size parameter",

451  &P);

452  check\_consistent\_sizes(function,

453  n, N, alpha, beta,

454  "Successes variable",

455  "Population size parameter",

456  "First prior sample size parameter",

457  "Second prior sample size parameter",

458  &P);

459

460  // Wrap arguments in vector views

461  VectorView<const T\_n> n\_vec(n);

462  VectorView<const T\_N> N\_vec(N);

463  VectorView<const T\_size1> alpha\_vec(alpha);

464  VectorView<const T\_size2> beta\_vec(beta);

465  size\_t size = max\_size(n, N, alpha, beta);

466

467  // Compute vectorized cdf\_log and gradient

468  using boost::math::lgamma;

469  using boost::math::digamma;

470

471  agrad::OperandsAndPartials<T\_size1, T\_size2>

472  operands\_and\_partials(alpha, beta);

473

474  // Explicit return for extreme values

475  // The gradients are technically ill-defined, but treated as neg infinity

476  for (size\_t i = 0; i < stan::length(n); i++) {

477  if (value\_of(n\_vec[i]) <= 0)

478  return operands\_and\_partials.to\_var(0.0);

479  }

480

481  for (size\_t i = 0; i < size; i++) {

482  // Explicit results for extreme values

483  // The gradients are technically ill-defined, but treated as zero

484  if (value\_of(n\_vec[i]) >= value\_of(N\_vec[i])) {

485  return operands\_and\_partials.to\_var(stan::math::negative\_infinity());

486  }

487

488  const double n\_dbl = value\_of(n\_vec[i]);

489  const double N\_dbl = value\_of(N\_vec[i]);

490  const double alpha\_dbl = value\_of(alpha\_vec[i]);

491  const double beta\_dbl = value\_of(beta\_vec[i]);

492

493  const double mu = alpha\_dbl + n\_dbl + 1;

494  const double nu = beta\_dbl + N\_dbl - n\_dbl - 1;

495

496  const double F = stan::math::F32(1, mu, -N\_dbl + n\_dbl + 1, n\_dbl + 2,

497  1 - nu, 1);

498

499  double C = lgamma(nu) - lgamma(N\_dbl - n\_dbl);

500  C += lgamma(mu) - lgamma(n\_dbl + 2);

501  C += lgamma(N\_dbl + 2) - lgamma(N\_dbl + alpha\_dbl + beta\_dbl);

502  C = std::exp(C);

503

504  C \*= F / boost::math::beta(alpha\_dbl, beta\_dbl);

505  C /= N\_dbl + 1;

506

507  const double Pi = C;

508

509  P += log(Pi);

510

511  double dF[6];

512  double digammaOne = 0;

513  double digammaTwo = 0;

514

515  if ( (!is\_constant\_struct<T\_size1>::value)

516  || (!is\_constant\_struct<T\_size2>::value) ) {

517  digammaOne = digamma(mu + nu);

518  digammaTwo = digamma(alpha\_dbl + beta\_dbl);

519  stan::math::gradF32(dF, 1, mu, -N\_dbl + n\_dbl + 1, n\_dbl + 2,

520  1 - nu, 1);

521  }

522  if (!is\_constant\_struct<T\_size1>::value) {

523  const double g

524  = - C \* (digamma(mu) - digammaOne + dF[1] / F

525  - digamma(alpha\_dbl) + digammaTwo);

526  operands\_and\_partials.d\_x1[i] -= g / Pi;

527  }

528  if (!is\_constant\_struct<T\_size2>::value) {

529  const double g

530  = - C \* (digamma(nu) - digammaOne - dF[4] / F - digamma(beta\_dbl)

531  + digammaTwo);

532  operands\_and\_partials.d\_x2[i] -= g / Pi;

533  }

534  }

535

536  return operands\_and\_partials.to\_var(P);

537  }

538

539  template <class RNG>

540  inline int

541  beta\_binomial\_rng(const int N,

542  const double alpha,

543  const double beta,

544  RNG& rng) {

545

546  static const char\* function = "stan::prob::beta\_binomial\_rng(%1%)";

547

548  using stan::math::check\_positive\_finite;

549  using stan::math::check\_nonnegative;

550

551  check\_nonnegative(function, N, "Population size parameter", (double\*)0);

552  check\_positive\_finite(function, alpha,

553  "First prior sample size parameter",

554  (double\*)0);

555  check\_positive\_finite(function, beta,

556  "Second prior sample size parameter",

557  (double\*)0);

558

559  double a = stan::prob::beta\_rng(alpha, beta, rng);

560  while(a > 1 || a < 0)

561  a = stan::prob::beta\_rng(alpha, beta, rng);

562  return stan::prob::binomial\_rng(N, a, rng);

563  }

564 }

565  }

566 #endif

stan::agrad::lbeta

fvar< T > lbeta(const fvar< T > &x1, const fvar< T > &x2)

**Definition:** lbeta.hpp:16

beta.hpp

traits.hpp

stan::agrad::OperandsAndPartials::to\_var

T\_return\_type to\_var(double logp)

**Definition:** partials\_vari.hpp:138

stan::math::check\_positive\_finite

bool check\_positive\_finite(const char \*function, const T\_y &y, const char \*name, T\_result \*result)

**Definition:** check\_positive\_finite.hpp:12

error\_handling.hpp

stan::length

size\_t length(const T &)

**Definition:** traits.hpp:159

stan::math::binomial\_coefficient\_log

boost::math::tools::promote\_args< T\_N, T\_n >::type binomial\_coefficient\_log(const T\_N N, const T\_n n)

Return the log of the binomial coefficient for the specified arguments.

**Definition:** binomial\_coefficient\_log.hpp:63

stan::DoubleVectorView

DoubleVectorView allocates double values to be used as intermediate values.

**Definition:** traits.hpp:358

stan::prob::beta\_binomial\_ccdf\_log

return\_type< T\_size1, T\_size2 >::type beta\_binomial\_ccdf\_log(const T\_n &n, const T\_N &N, const T\_size1 &alpha, const T\_size2 &beta)

**Definition:** beta\_binomial.hpp:428

partials\_vari.hpp

stan::agrad::value\_of

T value\_of(const fvar< T > &v)

Return the value of the specified variable.

**Definition:** value\_of.hpp:16

constants.hpp

stan::is\_vector

**Definition:** traits.hpp:83

stan::agrad::lgamma

fvar< T > lgamma(const fvar< T > &x)

**Definition:** lgamma.hpp:15

stan::prob::binomial\_rng

int binomial\_rng(const int N, const double theta, RNG &rng)

**Definition:** binomial.hpp:470

stan::agrad::OperandsAndPartials

A variable implementation that stores operands and derivatives with respect to the variable...

**Definition:** partials\_vari.hpp:76

stan::prob::beta\_binomial\_rng

int beta\_binomial\_rng(const int N, const double alpha, const double beta, RNG &rng)

**Definition:** beta\_binomial.hpp:541

traits.hpp

stan::return\_type::type

boost::math::tools::promote\_args< typename scalar\_type< T1 >::type, typename scalar\_type< T2 >::type, typename scalar\_type< T3 >::type, typename scalar\_type< T4 >::type, typename scalar\_type< T5 >::type, typename scalar\_type< T6 >::type >::type type

**Definition:** traits.hpp:406

stan::is\_constant\_struct

Metaprogram to determine if a type has a base scalar type that can be assigned to type double...

**Definition:** traits.hpp:57

stan::math::value\_of

double value\_of(const T x)

Return the value of the specified scalar argument converted to a double value.

**Definition:** value\_of.hpp:24

stan::prob::include\_summand

Template metaprogram to calculate whether a summand needs to be included in a proportional (log) prob...

**Definition:** traits.hpp:35

stan::math::lbeta

boost::math::tools::promote\_args< T1, T2 >::type lbeta(const T1 a, const T2 b)

Return the log of the beta function applied to the specified arguments.

**Definition:** lbeta.hpp:59

stan::agrad::OperandsAndPartials::d\_x2

VectorView< double \*, is\_vector< T2 >::value, is\_constant\_struct< T2 >::value > d\_x2

**Definition:** partials\_vari.hpp:83

binomial.hpp

stan::math::gradF32

void gradF32(double \*g, double a, double b, double c, double d, double e, double z, double precision=1e-6)

**Definition:** internal\_math.hpp:50

stan::math::check\_nonnegative

bool check\_nonnegative(const char \*function, const T\_y &y, const char \*name, T\_result \*result)

**Definition:** check\_nonnegative.hpp:52

stan::prob::beta\_binomial\_cdf

return\_type< T\_size1, T\_size2 >::type beta\_binomial\_cdf(const T\_n &n, const T\_N &N, const T\_size1 &alpha, const T\_size2 &beta)

**Definition:** beta\_binomial.hpp:191

stan::math::F32

double F32(double a, double b, double c, double d, double e, double z, double precision=1e-6)

**Definition:** internal\_math.hpp:14

stan::math::check\_consistent\_sizes

bool check\_consistent\_sizes(const char \*function, const T1 &x1, const T2 &x2, const char \*name1, const char \*name2, T\_result \*result)

**Definition:** check\_consistent\_sizes.hpp:13

stan::max\_size

size\_t max\_size(const T1 &x1, const T2 &x2)

**Definition:** traits.hpp:191

stan::prob::beta\_rng

double beta\_rng(const double alpha, const double beta, RNG &rng)

**Definition:** beta.hpp:593

internal\_math.hpp

stan::prob::beta\_binomial\_cdf\_log

return\_type< T\_size1, T\_size2 >::type beta\_binomial\_cdf\_log(const T\_n &n, const T\_N &N, const T\_size1 &alpha, const T\_size2 &beta)

**Definition:** beta\_binomial.hpp:314

stan::prob::beta\_binomial\_log

return\_type< T\_size1, T\_size2 >::type beta\_binomial\_log(const T\_n &n, const T\_N &N, const T\_size1 &alpha, const T\_size2 &beta)

**Definition:** beta\_binomial.hpp:28

stan::agrad::OperandsAndPartials::d\_x4

VectorView< double \*, is\_vector< T4 >::value, is\_constant\_struct< T4 >::value > d\_x4

**Definition:** partials\_vari.hpp:85

stan::math::size

int size(const std::vector< T > &x)

**Definition:** size.hpp:11

stan::agrad::binomial\_coefficient\_log

fvar< T > binomial\_coefficient\_log(const fvar< T > &x1, const fvar< T > &x2)

**Definition:** binomial\_coefficient\_log.hpp:16

stan::agrad::digamma

fvar< T > digamma(const fvar< T > &x)

**Definition:** digamma.hpp:16

value\_of.hpp

stan::agrad::OperandsAndPartials::d\_x1

VectorView< double \*, is\_vector< T1 >::value, is\_constant\_struct< T1 >::value > d\_x1

**Definition:** partials\_vari.hpp:82

stan::agrad::OperandsAndPartials::d\_x3

VectorView< double \*, is\_vector< T3 >::value, is\_constant\_struct< T3 >::value > d\_x3

**Definition:** partials\_vari.hpp:84

binomial\_coefficient\_log.hpp

lbeta.hpp

constants.hpp

stan::agrad::log

fvar< T > log(const fvar< T > &x)

**Definition:** log.hpp:15

stan::VectorView

VectorView is a template metaprogram that takes its argument and allows it to be used like a vector...

**Definition:** traits.hpp:275

stan::agrad::exp

fvar< T > exp(const fvar< T > &x)

**Definition:** exp.hpp:16

stan::math::negative\_infinity

double negative\_infinity()

Return negative infinity.

**Definition:** constants.hpp:123


---

[ Stan Home Page ]

*© 2011–2014,
Stan Development Team.*
